# Identification of mulberry leaf flavonoids and evaluating their protective effects on H_2_O_2_-induced oxidative damage in equine skeletal muscle satellite cells

**DOI:** 10.3389/fmolb.2024.1353387

**Published:** 2024-04-08

**Authors:** Xinzhuang Zhang, Aopan Geng, Di Cao, Manglai Dugarjaviin

**Affiliations:** lnner Mongolia Key Laboratory of Equine Science Research and Technology Innovation, College of Animal Science and Technology, Inner Mongolia Agricultural University, Hohhot, China

**Keywords:** mulberry leaf flavonoids, metabolomic, oxidative stress, Nrf2, equine skeletal muscle satellite cells

## Abstract

**Introduction:** Horses are susceptible to oxidative stress during strenuous endurance exercise, leading to muscle fatigue and damage. Mulberry leaf flavonoids (MLFs) possess significant antioxidant properties. However, the antioxidant efficacy of MLFs can be influenced by the extraction process, and their impact on H_2_O_2_-induced oxidative stress in equine skeletal muscle satellite cells (ESMCs) remains unexplored.

**Methods:** Our study employed three extraction methods to obtain MLFs: ultrasound-assisted extraction (CEP), purification with AB-8 macroporous resin (RP), and n-butanol extraction (NB-EP). We assessed the protective effects of these MLFs on H_2_O_2_-induced oxidative stress in ESMCs and analyzed the MLF components using metabolomics.

**Results:** The results revealed that pre-treatment with MLFs dose-dependently protected ESMCs against H_2_O_2_-induced oxidative stress. The most effective concentrations were 0.8 mg/mL of CEP, 0.6 mg/mL of RP, and 0.6 mg/mL of NB-EP, significantly enhancing EMSC viability (*p* < 0.05). These optimized MLF concentrations promoted the GSH-Px, SOD and T-AOC activities (*p* < 0.05), while reducing MDA production (*p* < 0.05) in H_2_O_2_-induced ESMCs. Furthermore, these MLFs enhanced the gene expression, including *Nrf2* and its downstream regulatory genes (*TrxR1*, *GPX1*, *GPX3*, *SOD1*, and *SOD2*) (*p* < 0.05). In terms of mitochondrial function, ESMCs pre-treated with MLFs exhibited higher basal respiration, spare respiratory capacity, maximal respiration, ATP-linked respiration compared to H_2_O_2_-induced ESMCs (*p* < 0.05). Additionally, MLFs enhanced cellular basal glycolysis, glycolytic reserve, and maximal glycolytic capacity (*p* < 0.05). Metabolomics analysis results revealed significant differences in mulberrin, kaempferol 3-O-glucoside [X-Mal], neohesperidin, dihydrokaempferol, and isobavachalcone among the three extraction processes (*p* < 0.05).

**Discussion:** Our study revealed that MLFs enhance antioxidant enzyme activity, alleviate oxidative damage in ESMCs through the activation of the Nrf2 pathway, and improve mitochondrial respiration and cell energy metabolism. Additionally, we identified five potential antioxidant flavonoid compounds, suggesting their potential incorporation into the equine diet as a strategy to alleviate exercise-induced oxidative stress.

## 1 Introduction

Horse racing is a vital economic industry in developed nations like the United States and Japan (Rankins and Malinowski, 2020). Horses’ athletic performance plays a crucial role in this industry, driven by increased energy demands during locomotion, which heightens mitochondrial respiration and muscle oxygen consumption. Approximately 5% of the overall oxygen is transformed to free radicals, such as hydroxyl radicals, superoxide anions, and hydrogen peroxide (Avellini et al., 1999). Excessive free radical production surpassing horses’ natural antioxidant defense capacity, results in oxidative stress. Various horse types, such as endurance horses (Hargreaves et al., 2002; Marlin et al., 2002; Williams et al., 2004; Brkljača Bottegaro et al., 2018), show jumpers, dressage horses (Muñoz-Escassi et al., 2006; Soares et al., 2011), Pentathlon Horses (Balogh et al., 2001), Marremmana racehorses (Chiaradia et al., 1998), Thoroughbred racehorses (Ishida et al., 1999; White et al., 2001), and Standardbred trotters (Sara et al., 2012), can experience oxidative stress during moderate to high-intensity exercises (Mills et al., 1996; Avellini et al., 1999; de Moffarts et al., 2006). Exercise-induced free radicals can negatively impact cellular functions, potentially causing genomic instability due to DNA damage and inefficient repair. Similarly, lipid and protein damage can also disrupt cellular homeostasis (Davison and McClean, 2022). Heightened levels of ROS result in muscle fatigue and damage, thus impairing horses’ athletic performance and overall wellbeing (Powers and Jackson, 2008).

Exogenous antioxidant supplements like vitamin E (Nemec Svete et al., 2021), vitamin C (Garcia et al., 2022), and selenium (Brummer et al., 2013), are widely utilized to mitigate oxidative stress, muscle damage, and inflammation in athletic horses, enhancing exercise performance. Recent years have seen extensive attention to the development and in-depth study of exogenous antioxidant supplement products. Flavonoids are a class of plant-derived dietary compounds that are found abundantly in fruits, vegetables, and herbal sources (Park et al., 2013; Shen et al., 2022). Previous studies have reported that flavonoids in other plants show high antioxidant activity and are able to neutralize free radicals (Wang et al., 2017; Wan et al., 2018b). Based on this evidence, we hypothesize that flavonoids could reduce the damage of oxidative stress on horse muscle tissue. Mulberry leaves (*Morus alba* L.), a high-quality animal feed resource, possess pharmacological effects, including antioxidant, blood glucose reduction, cholesterol regulation (impacting lipid metabolism), immune modulation, and anti-inflammatory properties (Ma et al., 2022). Various *in vitro* and *in vivo* experiments have linked the excellent antioxidant properties of mulberry leaves to their flavonoid compounds, particularly rutin (Katsube et al., 2006; Shilpi Singh et al., 2019), quercetin (Wang et al., 2013; Lin et al., 2022b), and astragalin (Choi et al., 2013; Yang et al., 2014). These flavonoid compounds are proven effective antioxidants and xanthine oxidase inhibitors (Wan et al., 2018a). Moreover, Wen et al. (Wen et al., 2020) identified a novel flavonoid in mulberry leaves, Morachalone D, which enhances the antioxidant defense system by upregulating the gene expression of *Nrf2*, *CAT*, *SOD2*, *GPx4*, *SLC7A11*, and *HMOX1*. Meng et al. (Meng et al., 2020) discovered methanol extracts from mulberry leaves enhance glucose metabolism and mitochondrial function through the AMPK-PGC-1α signaling pathway. However, the composition of mulberry leaf flavonoids (MLFs) is influenced by various extraction processes. For instance, Lin et al. (Lin et al., 2022a) extracted MLFs using ethanol reflux and AB-8 macroporous resin, yielding predominantly quercetin, kaempferol, and their derivatives. Conversely, Zheng et al. (Zheng et al., 2022) used ultrasound-assisted and D101 macroporous resin to extract MLFs, yielding mainly hesperidins, rutin, hyperoside, isoquercetin, cyanidin, and their derivatives. Currently, it remains unclear which flavonoid compound exhibits the most potent antioxidant activity in alleviating oxidative stress during equine exercise, necessitating further identification of specific MLF components. Ultrasound-assisted extraction has many advantages in extracting different compounds from plant materials, including increased yield, energy savings and reduced time (Žlabur et al., 2020). This makes it a basic and affordable strategy, especially suitable for extracting flavonoids, which can effectively increase the yield of plant extraction (Tungmunnithum et al., 2021). Organic solvents (such as methanol, ethanol, and n-butanol) are the most commonly used and efficient solvents for extracting flavonoids from plants (Venkatesan et al., 2019; Wu et al., 2022). ESMCs, small adult stem cells surrounding muscle fibers, play a pivotal role in promoting equine muscle regeneration and investigating endogenous oxidative stress (Dumont et al., 2015). Previous studies have reported that the seemingly beneficial effect of sustained flavonoid intake on muscle mass is based on two main effects: 1) stimulating mitochondrial biogenesis, resulting in increased ATP production; and 2) reducing the production of reactive oxygen species through the action of chemical scavengers, thereby improving muscle performance (Mukai et al., 2013; Gibellini et al., 2015; Munguía et al., 2022). This study evaluates MLFs’ potential in alleviating H_2_O_2_-induced oxidative stress in ESMCs. MLFs were extracted using three methods: ultrasound-assisted extraction (CEP), AB-8 macroporous resin purification (RP), and n-butanol extraction (NB-EP). Metabolomic analysis identified MLF components. Employing ESMCs as an *in vitro* model, we assessed cell morphology, antioxidant enzyme activity, antioxidant gene expression, and energy metabolism to explore the protective effects of MLFs.

## 2 Materials and methods

### 2.1 Experimental materials

Recently harvested mulberry leaf samples were collected from the Jinhua planting base in Zhejiang province, washed, and oven-dried for 24 h until a constant weight was reached. The dried leaves were then finely powdered using an ultra-disintegrator.

We sourced AB-8 macroporous resin from Ruida Henghui Company. Additionally, the following analytical pure chemicals were obtained from Thermo Company: H2O2, rutin, HCl, sodium hydroxide, n-butanol, sodium nitrite, aluminum nitrate, ethanol, methanol, and acetonitrile. Cell culture reagents were all purchased from Gibco Co., Ltd. Commercial assay kits for plasma glutathione peroxidase (GSH-Px), superoxide dismutase (SOD), total antioxidant capacity (T-AOC), and malondialdehyde (MDA) determination were purchased from Najingjiancheng Biotechnology Co. Ltd. Seahorse XF 1.0M glucose solution, Seahorse XF 100 mM pyruvic solution, Seahorse XF RPMI medium, Seahorse XF 200 mM glutamine solution were acquired from Agilent Company.

### 2.2 Extraction of MLFs

#### 2.2.1 Ultrasound-assisted extraction of MLFs

To extract MLFs, an 80 kHz ultrasonic bath (P300H, Elmasonic, Germany) was employed. First, mix 5 g of mulberry leaf powder with 150 mL of 55% ethanol, while maintaining the water bath at 70°C (±1.0 °C). The entire extraction process lasted 17 min. The resulting slurry underwent centrifugation at 8,000 rpm for 10 min to collect the supernatant. This supernatant was concentrated via a rotary evaporator (RE52, Yarong, China) under vacuum at 55 °C. The resulting extract was frozen at −80 °C overnight, followed by freeze-drying in a Lyomicon55 (Coolvacuum, Spain). Finally, the microwave-assisted crude extraction of MLFs’ lyophilized powder (CEP) was obtained and reserved for use.

#### 2.2.2 AB-8 macroporous resin for MLFs purification

The AB-8 macroporous resin was sequentially immersed in 5% HCl and 5% NaOH for 6 h, with distilled water rinsing after each step for pH neutralization. Subsequently, it was immersed in 95% ethanol for 12 h and rinsed until ethanol was eliminated. An initial resin load of 10 g was used. After optimization tests for static adsorption and desorption, a 0.05 g/mL loading solution concentration and a 55% ethanol concentration in the eluent were chosen. In dynamic adsorption and desorption optimization experiments, a 60 mL loading liquid volume and a 30 mL elution liquid volume were selected. Following these conditions, CEP was purified. The separated effluent was evaporated, frozen at −80 °C overnight, and freeze-dried the next day. Finally, this process yielded the purified MLFs (RP).

#### 2.2.3 Extraction of MLFs with n-butanol

The CEP was extracted using n-butyl alcohol at a 0.05 g/mL solid-liquid ratio. Subsequently, the extract underwent concentration through a rotary evaporator, resulting in the final product (NB-EP) following freeze-drying.

### 2.3 Isolation and culture of ESMCs

Semitendinosus muscle samples were obtained from a healthy 6-month-old colt at a commercial abattoir. The procedures for slaughter and sample collection adhered to Animal Welfare Committee guidelines at Inner Mongolia Agricultural University (Approval Code: 2020102). Primary equine satellite cells were isolated and cultured as previously described (Xing et al., 2023). Briefly, semitendinosus muscle samples from a healthy 6-month-old foal were immersed in cold Dulbecco’s Phosphate-Buffered Saline, washed, and had connective tissue removed. The muscle tissue was then minced in 0.2% type I collagenase solution, followed by shaking table digestion in a biochemical incubator at 37 °C for 1 h. After centrifugation at 3,000 rpm for 5 min and removing the supernatant, the cells passed through two filters with pore sizes of 100 μm and 70 μm, respectively. Subsequently, another centrifugation at 3,000 rpm for 5 min was performed, and the supernatant was then discarded. The resulting precipitate was resuspended in a proliferation medium comprising Dulbecco’s Modified Eagle Medium (DMEM), 10% Fetal Bovine Serum (FBS), 1% antibiotics, and 2.5 ng/mL bFGF. To enhance the purification of ESMCs and minimize fibroblast contamination, a differential adhesion technique was employed. The suspended cells were initially seeded onto a matrix-glue-coated Petri dish for 2 h. Then, the supernatant was moved to a new matrix-glue-coated Petri dish. After adding proliferation medium, the sample was incubated in a 5% CO_2_ environment at 37 °C, with the growth medium refreshed every 2 days. The purification steps was iterated to achieve over 90% purity for ESMCs. During proliferation, when cells were 90% confluence, they were treated with accutase and placed in FBS containing 10% DMSO(dimethyl sulfoxide). The cells were frozen in liquid nitrogen for future utilization. Finally, the ESMCs were cultured in proliferation medium (PM), consisting of 89% DMEM, 10% FBS, and 1% antibiotics (penicillin and streptomycin). PM was pre-filtered with a 0.22 µm filter.

### 2.4 Experimental design

The ESMCs were categorized into four groups: 1) Normal control (NC): Cells cultured in PM for 24 h, 2) Protection group (CEP, RP, and NB-EP groups): Cells were pre-protected in CEP, RP, and NB-EP solution with PM solutions at 0.2 mg/mL, 0.4 mg/mL, 0.6 mg/mL, 0.8 mg/mL, and 1.0 mg/mL, respectively for 24 h, 3) Damaged group (H_2_O_2_): Cells cultured in PM for 24 h, then treated with 600 μM H_2_O_2_ for 6 h, 4) Experimental group (CEP + H_2_O_2_, RP + H_2_O_2_, and NB-EP + H_2_O_2_ groups): Cells were pre-protected in CEP, RP, and NB-EP solutions with PM solutions at 0.2 mg/mL, 0.4 mg/mL, 0.6 mg/mL, 0.8 mg/mL, and 1.0 mg/mL for 24 h, respectively, and then treated with 600 μM H_2_O_2_ for 6 h. Each treatment group underwent five replicates.

### 2.5 Effects of MLFs on H_2_O_2_-induced oxidative stress in ESMCs

#### 2.5.1 Cells morphological observation

Cells were examined and captured at 10× magnification using a Primovert microscope (Carl Zeiss, Germany).

#### 2.5.2 Cell viability

In 96-well plates, 100 μL of PM containing 2 × 10^3^ cells/mL was inoculated and conducted to different groups procedures as described above Cell Proliferation Rate was determined via MTT colorimetric assay. After treatment, 50 μL of 1 × MTT solution was added to each well, and the mixture was then incubated at 37 °C for 4 h. Following incubation, the supernatant was aspirated, and 150 μL of DMSO was added to dissolve formazan crystals. Measure the absorbance of the mixture at 570 nm using a microplate reader (EPOCH, BioTek, United States).

### 2.6 Effect of MLFs on antioxidant capacity in H_2_O_2_-induced ESMCs

To assess MLFs’ impact on antioxidant capacity in H_2_O_2_-induced ESMCs, GSH-Px, SOD, T-AOC, and MDA were measured using an assay kit.

### 2.7 Effect of MLFs on gene expression of antioxidant in H_2_O_2_-induced ESMCs

Based on the above-mentioned results, 0.8 mg/mL CEP, 0.6 mg/mL RP, and 0.6 mg/mL NB-EP groups were selected to examine MLFs’ impact on antioxidant gene expression in H_2_O_2_-induced ESMCs. Their mRNA levels for *Nrf2*, *SOD1*, *SOD2*, *GPX1*, *GPX3*, and *TrxR1* were assessed using real-time PCR. The total RNA was isolated from various treatments with the RNA kit (Ambion-1561, USA) and reverse-transcribed it into cDNA (TaKaRa, Dalian, China) following the manufacturer’s instructions. The primer sequences are shown in Table 1. The real-time PCR reaction system comprised 10 μL of TB Green Premix Ex Taq II, 6.4 μL of RNase-free water, 0.8 μL of a mix of forward and reverse primers, and 2 μL of cDNA template. The cDNA amplification was monitored using the CFX 96 Real-Time PCR detection system (Biorad, United States). Finally, relative gene expression levels were determined using the comparative 2^-△△Ct^ method, with *GAPDH* as the endogenous control.

**TABLE 1:** Primer sequences.

Genes name	Primer sequences (5′–3′)
*Nrf2-F*	CCA​GTA​CCA​GCA​ACG​GCA​T
*Nrf2-R*	TGT​TGT​GCT​TTC​AGG​GTG​GT
*SOD1- F*	TGC​TCA​CTT​TAA​TCC​TCT​GTC​G
*SOD1- R*	AAT​GCT​TTC​CCG​AGA​GTG​AG
*SOD2- F*	ACG​TGA​CTT​TGG​TTC​CTT​GG
*SOD2- R*	CGT​CCC​TGG​TCC​TTA​TTG​AAA​C
*GPX1- F*	ATC​AGG​AGA​ACG​CCA​AGA​AC
*GPX1- R*	TCA​CCT​CGC​ACT​TCT​CAA​AG
*GPX3- F*	GTC​TGG​TCA​TTC​TGG​GCT​TC
*GPX3- R*	CCG​TTC​ACA​TCC​CCT​TTC​TC
*TrxR1- F*	TTT​TGT​CAC​TCC​AAC​CCC​TC
*TrxR1- R*	TCG​ACA​TTC​CAT​CCG​TAG​TTT​C

### 2.8 Effect of MLFs on energy metabolism in H_2_O_2_-induced ESMCs

To investigate the impact of MLF pre-treatment on energy metabolism in ESMCs, real-time oxygen consumption rate (OCR) and extracellular acidification rate (ECAR) were measured using an Agilent Hippocampal XFP analyzer (S7802A, Agilent Technologies, United States). Cell suspensions exposed to oxidative stress were seeded in XFp cell culture plates and incubated in a 37 °C, 5% CO_2_ environment for cell adhesion for cell adhesion. After an overnight incubation, the growth medium was substituted with Seahorse detection solution (pH 7.4 ± 0.1), comprising 9.7 mL of XF basal medium, 100 μL of pyruvate, 100 μL of glutamine, and 100 μL of glucose, all pre-filtered through a 0.22 μm filter. Subsequently, cells were incubated at 37 °C without CO_2_ for 60 min. Concurrently, 20 μL of oligomycin (15 μM/mL) was added to well A of the probe plate, followed by 22 μL of FCCP (20 μM/mL) in well B. A blend containing a complex I inhibitor (rotenone) and complex III inhibitor (antimycin A) was introduced into well C of the probe plate. The XFp Cell Mito Stress Test Kit was used to measure OCR and ECAR.

### 2.9 Total flavonoids content determination

The total flavonoid content was quantified using a modified colorimetric method (Lin et al., 2022b). Briefly, NaNO_2_, Al (NO_3_)_3_, NaOH were sequentially reacted with MLFs, and their absorbance values were measured at 510 nm. Rutin was used as a standard to establish a standard curve to quantify its concentration (y = 1.9984x + 0.1096, *R* ^2^ = 0.9998), with y denoting rutin absorbance and × indicating rutin solution concentration.

### 2.10 Components of MLFs by UPLC-HRMS-based metabolomics

For chromatographic separation, a Thermo Vanquish system equipped with an ACQUITY UPLC^®^ BEH C18 (100 × 2.1 mm, 1.7 µm, Waters) column maintained at 40 °C was used. Analytes were eluted using a gradient with 0.1% formic acid in water (A2) and 0.1% formic acid in acetonitrile (B2) at a flow rate of 0.25 mL/min.

The ESI-MSn experiments were conducted using a Thermo Q Exactive mass spectrometer with a spray voltage of 3.5 kV and −2.5 kV in positive and negative modes, respectively. The analyzer scanned a mass range of m/z 81–1,000 at a mass resolution of 70,000 for the full scan. Data-dependent acquisition (DDA) MS/MS experiments utilized HCD scans with normalized collision energies of 30 eV, 50 eV, and 60 eV.

### 2.11 Identification, quantification, and data analysis of MLFs

To enhance reliability and intuition, the data was preprocessed using Pareto scaling (Mean-centering and scaled to Pareto variance, Par) before conducting multivariate statistical analysis. This experiment involved employing multivariate statistical methods to perform Principal Component Analysis (PCA), on three sample groups. The metabolites were initially identified, ensuring an accurate molecular weight (molecular weight error <30 ppm), and subsequently matched and annotated them using fragment information from MS/MS patterns in Metlin (http://metlin.scripps.edu), MoNA (https://mona.fiehnlab.ucdavis.edu/), and a custom standard substance database provided by Panomik Company. After identifying the molecular weights of all measured compounds, the metabolites were classified according to their chemical structures related to flavonoids.

### 2.12 Statistics

For statistical analysis, IBM SPSS 20.0 software (IBM SPSS, Chicago, IL) was used to conduct one-way ANOVA. GraphPad Prism 6 was utilized for creating diagrams. The data is presented as mean ± standard deviation, with statistical significance considered at *p* < 0.05.

## 3 Results

### 3.1 Total flavonoid content of the MLFs

As shown in Table 2, NB-EP extracted by n-butanol exhibited the highest total flavonoid content, RP extracted from A8 macroporous resin was intermediate, while CEP extracted by ultrasonic-assisted extraction had the lowest content of MLFs.

**TABLE 2:** Evaluation of the total flavonoid content.

MLFs	Content (%)
CEP	22.56 ± 0.26^b^
RP	37.94 ± 0.84^a^
NB-EP	40.96 ± 0.33^a^

Each value shows the mean ± SD (n = 3). Different superscript letters within the same column indicate statistically significant differences (*p* < 0.05).

### 3.2 Cytoprotective effect of MLFs on H_2_O_2_-induced oxidative stress in ESMCs

#### 3.2.1 Morphological observation of ESMCs

**FIGURE 1:**
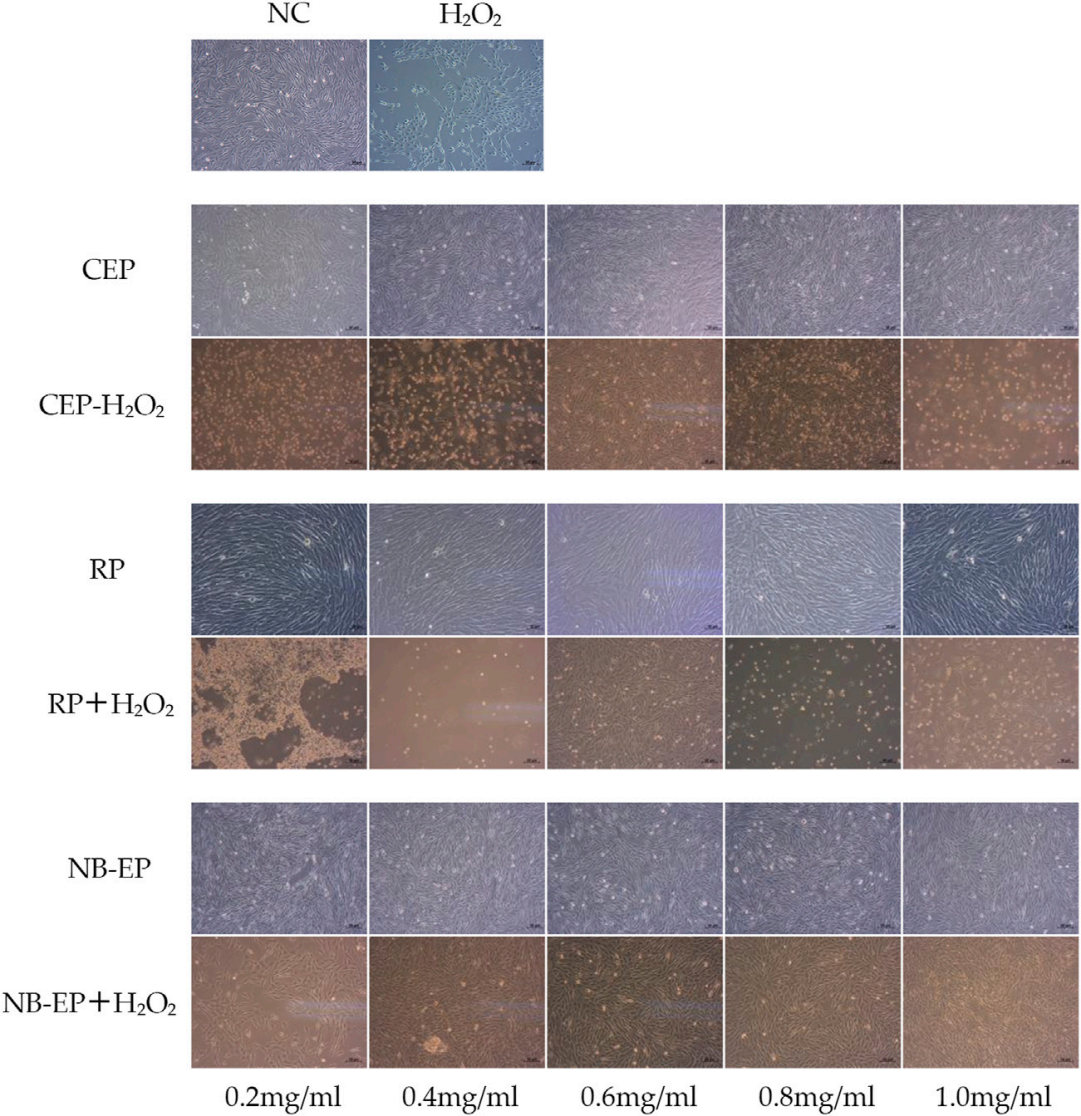
Morphology in H_2_O_2_-induced ESMCs treated with MLFs.

The study investigated the alleviating effect of MLFs with pre-treatment for H_2_O_2_-induced oxidative stress in ESMCs (Figure 1). The normal control group exhibited normal cell morphology, characterized by fusiform shape, abundant cytoplasm, strong cell membrane refractivity, and orderly cell arrangement. Upon exposure to CEP, RP, and NB-EP at of 0.2 mg/mL, 0.4 mg/mL, 0.6 mg/mL, 0.8 mg/mL, and 1.0 mg/mL, there were no significant changes in cell morphology compared to the normal control group. After H_2_O_2_ treatment, the damaged group showed significant cellular contraction, shifting from the typical fusiform shape of ESMCs to an elliptical form, and some cells were fragmented, which indicated severe damage. In the experimental group, pre-treatment with CEP, RP, and NB-EP at concentrations of 0.2 mg/mL, 0.4 mg/mL, 0.6 mg/mL, 0.8 mg/mL, and 1.0 mg/mL demonstrated a protective effect against H_2_O_2_-induced damage.

Overall, cell morphology analysis suggested that ESMCs did not exhibit significant cytotoxicity when exposed to CEP, RP, and NB-EP within the 0.2–1.0 mg/mL concentration range. However, the optimal protective concentrations varied among the three MLFs. CEP, RP, and NB-EP showed optimal protective effects at concentrations of 0.6–0.8 mg/mL, 0.6 mg/mL, and 0.4–0.8 mg/mL, respectively.

#### 3.2.2 Cell viability

**FIGURE 2:**
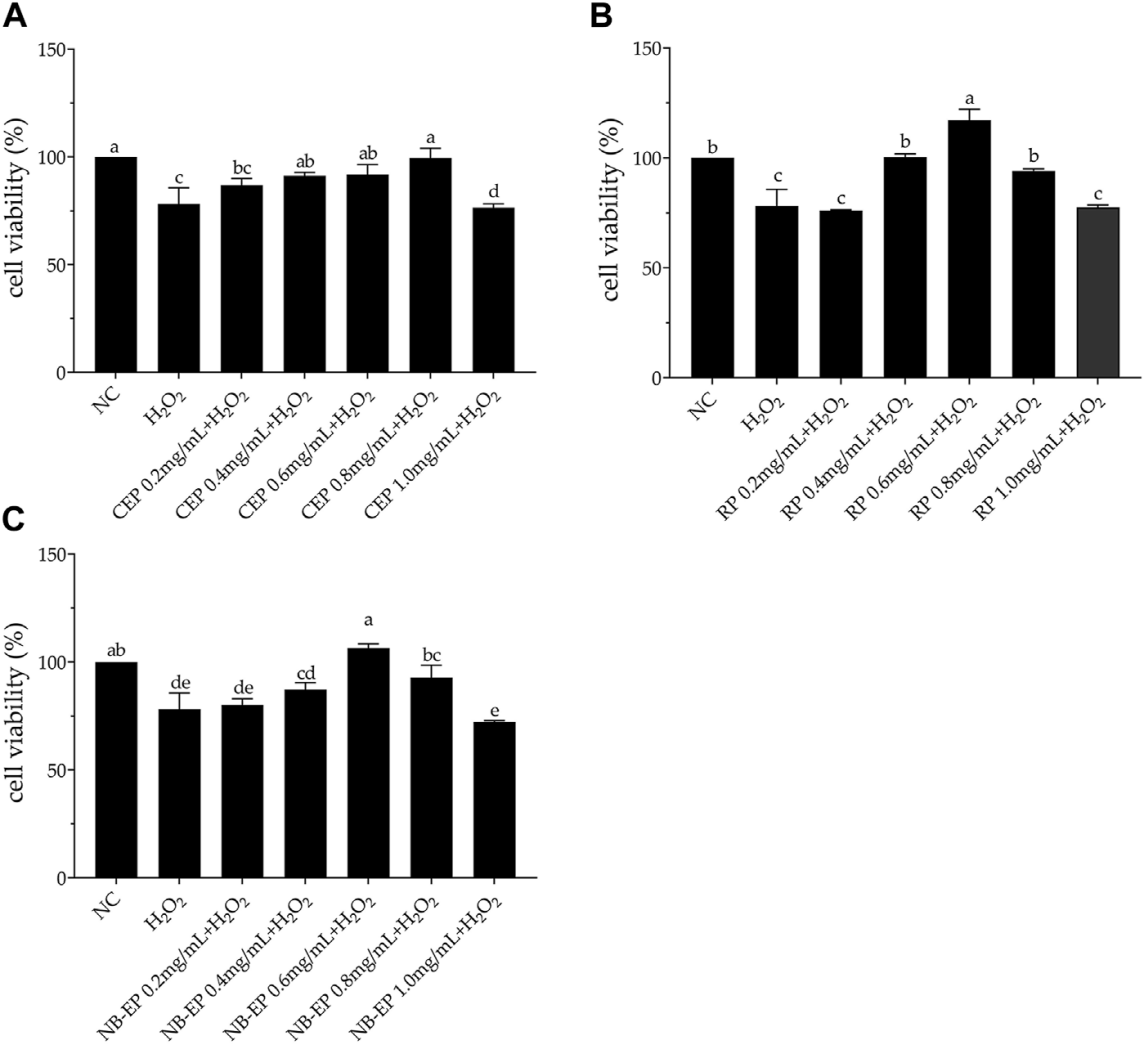
Effects of **(A)** CEP, **(B)** NB-EP, and **(C)** RP on the viability of ESMCs. Columns with different superscript letters are significantly different (*p* < 0.05).

We performed an MTT assay on EMSCs and compared them to the normal control group as a control (Figure 2). H_2_O_2_-treated cell viability was 78.16%, significantly lower than the normal control group (*p* < 0.05). Viability of ESMCs treated with 0.2, 0.4, 0.6, 0.8, and 1.0 mg/mL CEP was 86.78%, 91.33%, 91.87%, 99.50%, and 76.45%, respectively (Figure 2A). RP groups exhibited cell viabilities of 76.06%, 100.45%, 117.21%, 94.10%, and 77.46%, respectively (Figure 2B). Similarly, NB-EP groups showed cell viabilities of 80.09%, 87.37%, 106.41%, 92.83%, and 72.26%, respectively (Figure 2C). Optimal protective concentrations for safeguarding ESMCs against H_2_O_2_-induced damage were 0.8 mg/mL CEP, 0.6 mg/mL RP, and 0.6 mg/mL NB-EP. Additionally, we found that among the three MLF types, RP exhibited higher biological activity with a cell viability of 117.21%.

### 3.3 Effect of MLFs on the antioxidant properties on H_2_O_2_-induced ESMCs

**FIGURE 3:**
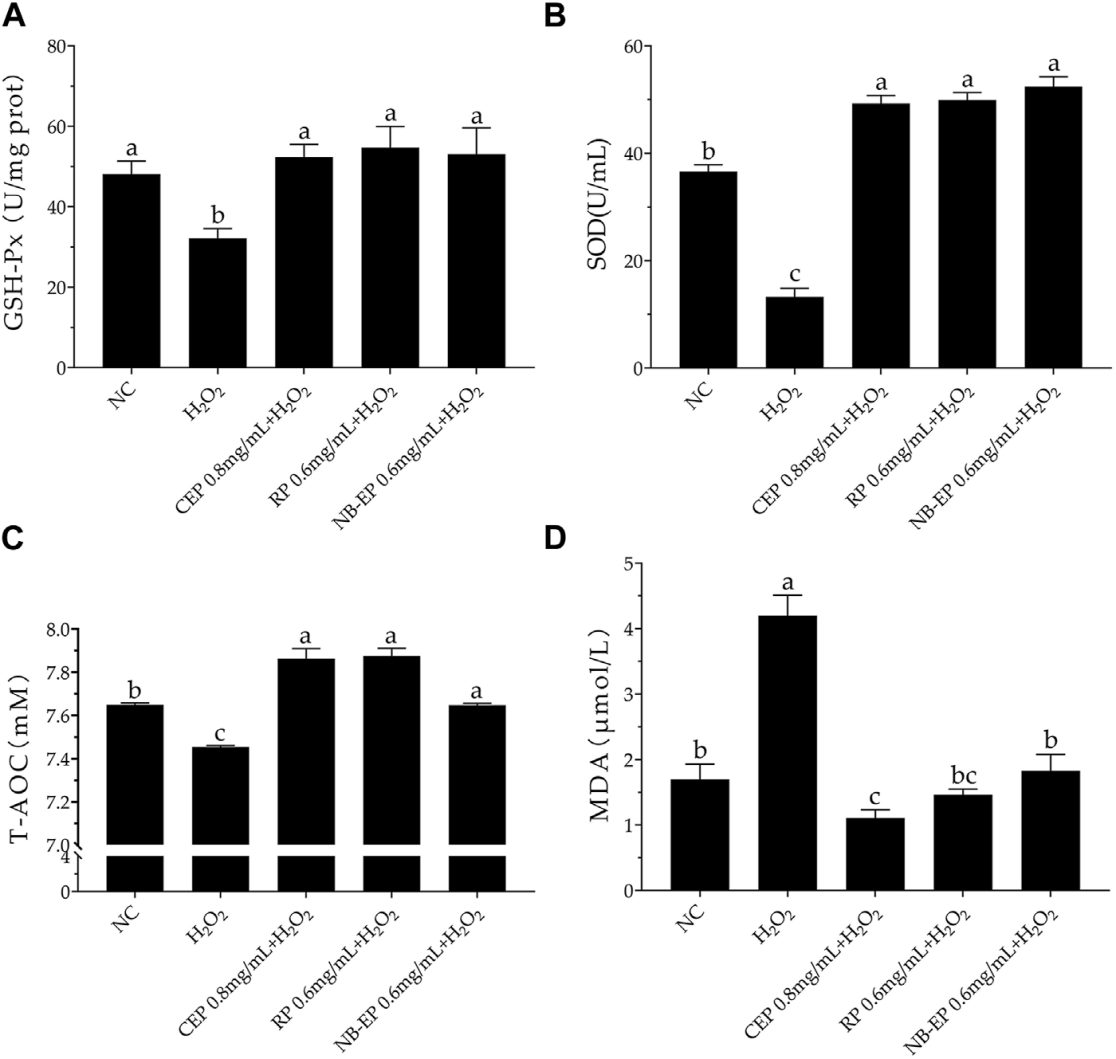
Effect of MLFs on Antioxidant properties in H_2_O_2_-induced ESMCs. **(A–D)** The GSH-Px, SOD, T-AOC, and MDA detected in ESMCs. Columns with different superscript letters are significantly different (*p* < 0.05).

Consistent with previous research, SOD, GSH-Px, and T-AOC activities significantly decreased upon H_2_O_2_ treatment compared to the normal control group (*p* < 0.05). Conversely, pre-treatment with different MLFs significantly increased the GSH-Px, SOD, and T-AOC activities as compared to the damaged group (*p* < 0.05), restoring them to normal cellular levels (Figures 3A–C). However, there were no significant differences in GSH-Px, SOD, and T-AOC indicators among the three MLFs.

The H_2_O_2_-treated damage group exhibited a significant accumulation of MDA levels, with a 4.2 μM increase compared to the 1.7 μM in the NC group, indicating severe lipid peroxidation in ESMCs. MLFs pre-treatment significantly reduced MDA levels compared to the damaged group, with the CEP group showing the lowest MDA concentration at 0.8 mg/mL (*p* < 0.05 compared to the normal control group).

### 3.4 Effect of MLFs on antioxidant gene expression on H_2_O_2_-induced ESMCs

**FIGURE 4:**
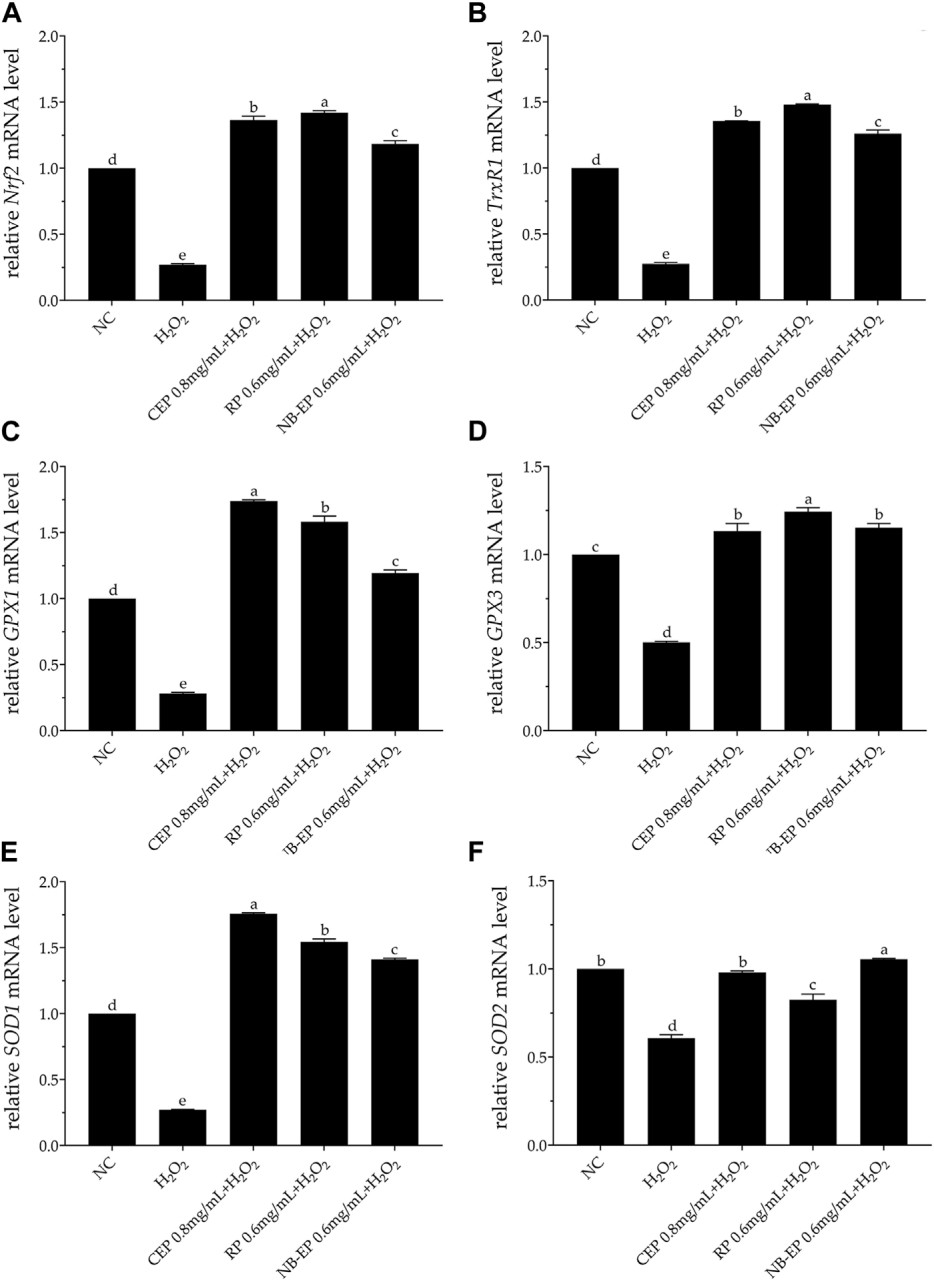
Effect of MLFs on *Nrf2* and its downstream regulatory genes in H_2_O_2_-treated ESMCs. qRT-PCR was used to measure the relative mRNA levels of **(A)** *Nrf2*, **(B)** *TrxR1*, **(C)** *GPX1*, **(D)** *GPX3*, **(E)** *SOD1*, and **(F)** *SOD2*. Columns with different superscript letters are significantly different (*p* < 0.05).

Our study suggests that MLFs alleviate H_2_O_2_-induced oxidative stress in ESMCs by boosting antioxidant enzyme activity through the Nrf2-ARE pathway. Therefore, we evaluated critical Nrf2-ARE pathway gene expression using qRT-PCR (Figure 4). The expression levels of *Nrf2*, *TrxR1*, *GPX1*, *GPX3*, *SOD1*, and *SOD2* genes of the damaged group were significantly inhibited compared with the normal control group (*p* < 0.05). MLF pre-treatment increased *Nrf2* and related downstream gene expression, including *TrxR1*, *GPX1*, *GPX3*, *SOD1*, and *SOD2*, compared to the damaged group (*p* < 0.05). Specifically, the expression levels of *GPX1* and *SOD1* in ESMCs (*p* < 0.05). The 0.6 mg/mL RP group exhibited the most significant *SOD2* upregulation (*p* < 0.05) compared to other experimental groups, while the 0.6 mg/mL NB-EP group showed the highest *Nrf2*, *GPX3*, and *SOD2* expression levels in ESMCs (*p* < 0.05).

### 3.5 Effect of MLFs on energy metabolism on H_2_O_2_-induced ESMCs

**FIGURE 5:**
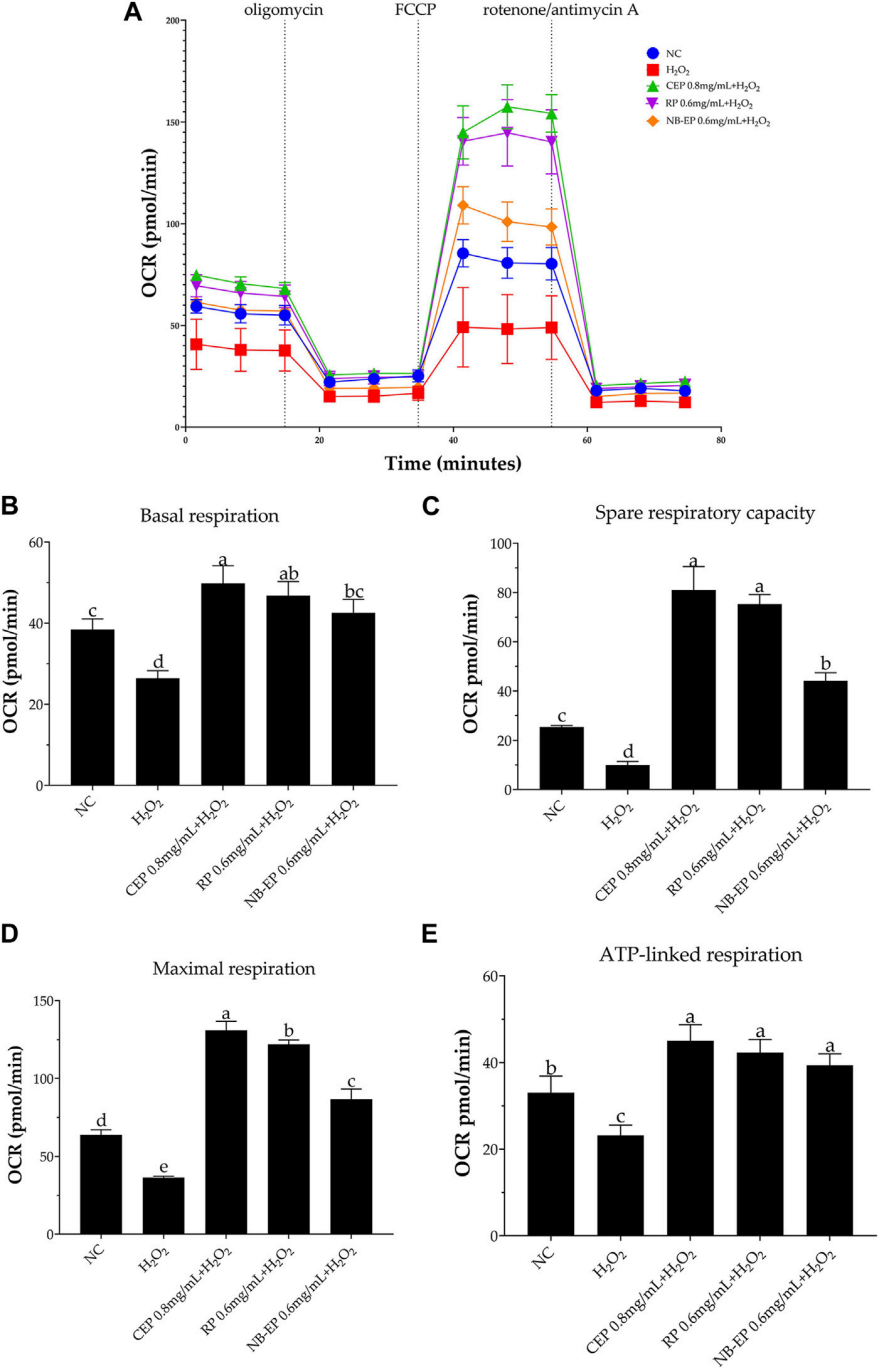
Effect of MLFs on Aerobic respiration in H_2_O_2_-induced ESMCs. **(A)** represented the respiratory curve of different groups, from which OCR is calculated to determine the **(B)** basal respiration, **(C)** spare respiratory capacity, **(D)** maximal respiration, and **(E)** ATP-linked respiration capacity. Data are presented as the means ± SD (n = 3). Columns with different superscript letters are significantly different (*p* < 0.05).

To investigate MLFs’ potential in preventing H_2_O_2_-induced loss of mitochondrial respiratory capacity, we assessed cellular bioenergetics in ESMCs cells using real-time OCR. We evaluated four stages of mitochondrial respiration: 1) basal respiration, 2) spare respiratory capacity, 3) maximal respiration, and 4) ATP-linked respiration. As shown in Figure 5A, H_2_O_2_ exposure led to a significantly decreased the OCR decrease in ESMCs, whereas pre-treatment with MLFs resulted in higher OCR levels (*p* < 0.05). In Figures 5B–F, the damaged group exhibited significant reductions of 31.27%, 60.79%, and 43.04%, respectively, in mitochondrial basal respiration, spare respiratory capacity, and maximal respiration compared to the NC group (*p* < 0.05). After pre-treatment with 0.8 mg/mL of CEP, 0.6 mg/mL of NB-EP, and 0.6 mg/mL of RP, the basal respiration of ESMCs significantly increased by 29.56%, 10.82%, and 21.83%, respectively, compared to the normal control group (*p* < 0.05). Additionally, their spare respiratory capacity significantly increased by 218.3%, 73.36%, and 192.54% (*p* < 0.05), and maximal respiration showed a notable increase of 104.81%, 35.76%, and 91.09% (*p* < 0.05). Furthermore, the ATP-linked respiration of cells pretreated with 0.8 mg/mL CEP, 0.6 mg/mL NB-EP, and 0.6 mg/mL RP increased to 136.36%, 119.33%, and 127.98% of normal cells (*p* < 0.05).

**FIGURE 6:**
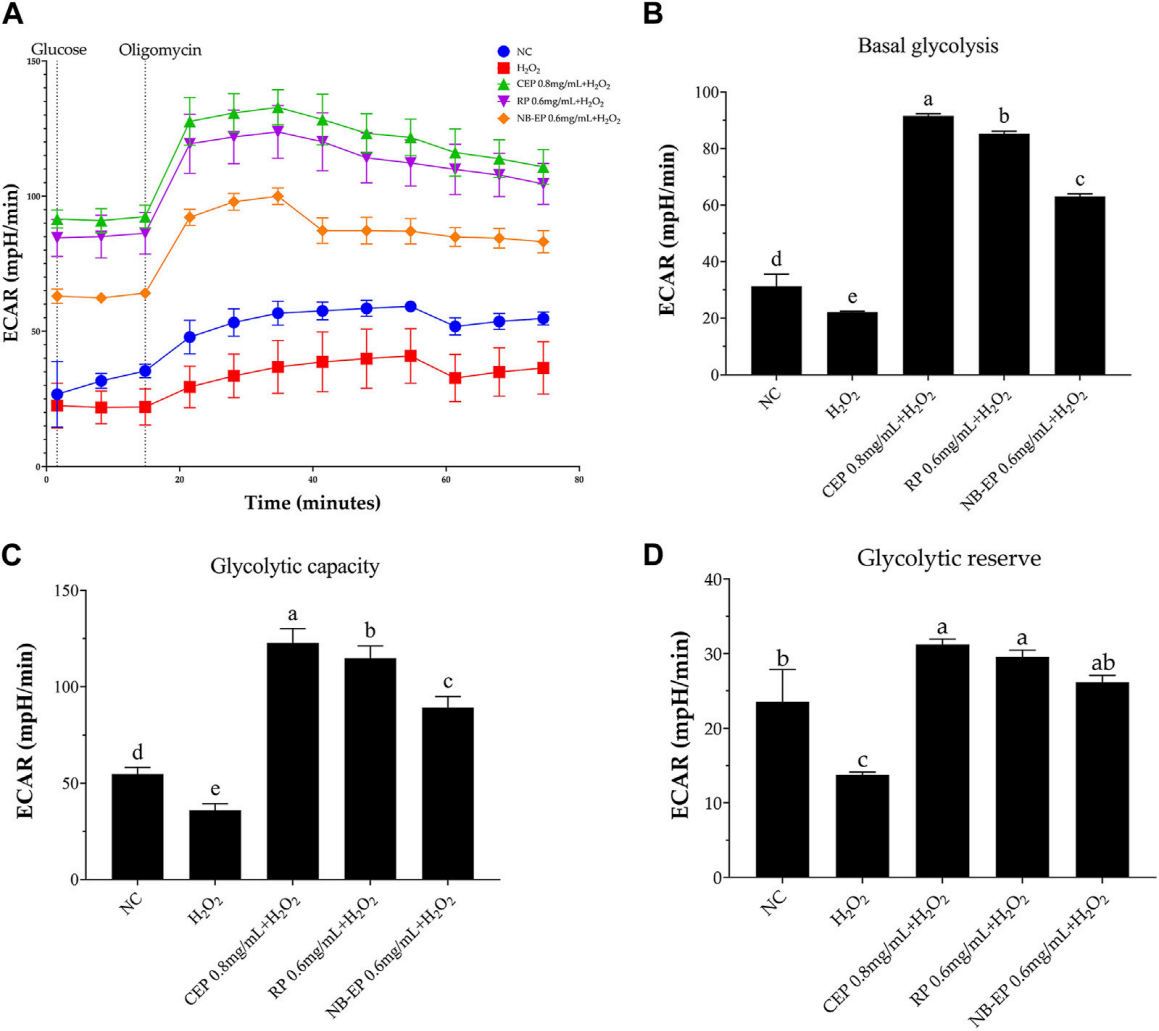
Effect of MLFs on glycolytic function in H_2_O_2_-induced ESMCs. **(A)** represented the extracellular acidification rate (ECAR) curve from which ECAR is calculated to determine **(B)** basal glycolysis, **(C)** Glycolytic capacity, and **(D)** Glycolytic reserve capacity. Columns with different superscript letters are significantly different (*p* < 0.05).

To further investigate MLFs’ impact on H_2_O_2_-induced glycolytic dysfunction, we measured ECAR and assessed basal glycolysis, glycolytic capacity, and glycolytic reserve. As shown in Figure 6A, ECAR significantly decreased after H_2_O_2_ exposure. However, pre-treatment with MLFs increased ECAR levels compared to the NC group (*p* < 0.05). The ESMCs in the damaged group exhibited a significant 31.27% decrease in basal glycolysis, a 60.79% decrease in glycolytic capacity, and a 43.04% decrease in glycolytic reserve compared to the NC group (*p* < 0.05). Following pre-treatment with 0.8 mg/mL of CEP, 0.6 mg/mL of NB-EP, and 0.6 mg/mL of RP, ESMCs showed significant increases in basal glycolysis (29.14%, 41.41%, and 34.41% respectively), glycolytic capacity (124.18%, 63.04%, and 109.64%), and glycolytic reserve (32.56%, 11.21%, and 25.64%) as compared to the normal control group (*p* < 0.05).

### 3.6 Identification of flavonoid compounds in MLFs

**FIGURE 7:**
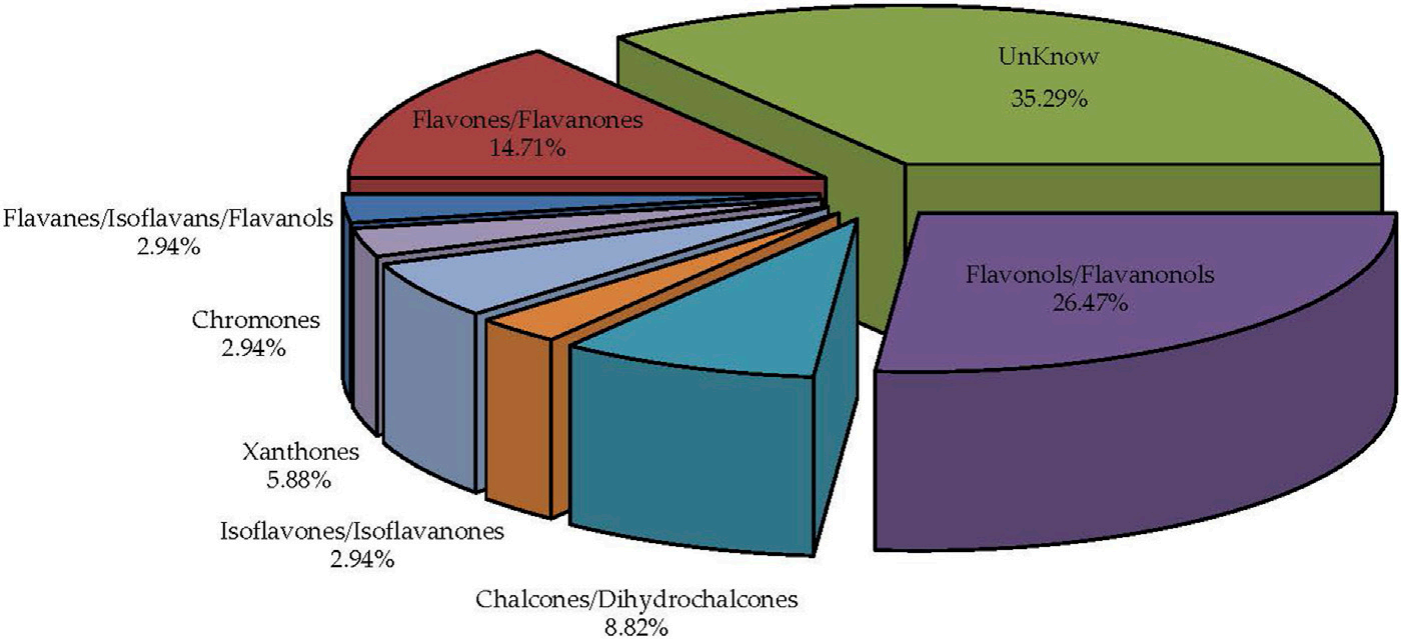
Comprehensive identification and classification chart of MLFs (n = 9).

The MLF categories (Figure 7) are organized based on flavonoid chemical structures, including Flavones/Flavanones, Bisflavones, Chalcones/Dihydrochalcones, Flavonols/Flavanonols, Chromones, Isoflavones/Isoflavanones, Flavanes/Isoflavans/Flavanols, Xanthones, Anthocyanins, and other categories. These findings are consistent with previously reported MLF components (Yang et al., 2014; Ma et al., 2022).

### 3.7 PCA analysis of MLFs

**FIGURE 8:**
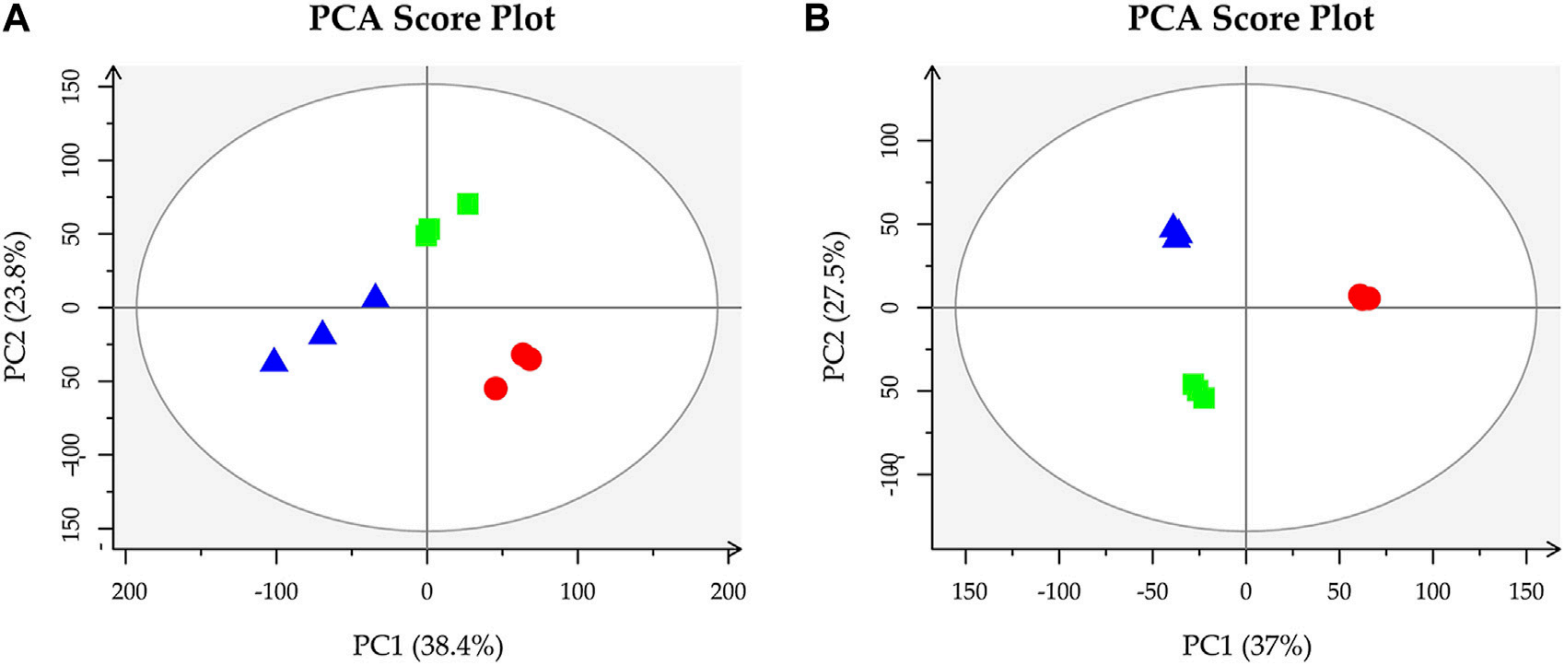
Perform principal component analysis on CEP (circle), RP (rectangle), and NB-EP (triangle) (n = 3). **(A, B)** stand for positive and negative ion modes, respectively.

PCA revealed non-overlapping positions for MLFs extracted using three different methods (Figure 8). This demonstrates that different extraction techniques can impact MLF constituents, ensuring the basis for subsequent metabolomics bioinformatics analysis.

### 3.8 Differential flavonoids among three types of MLFs

**FIGURE 9:**
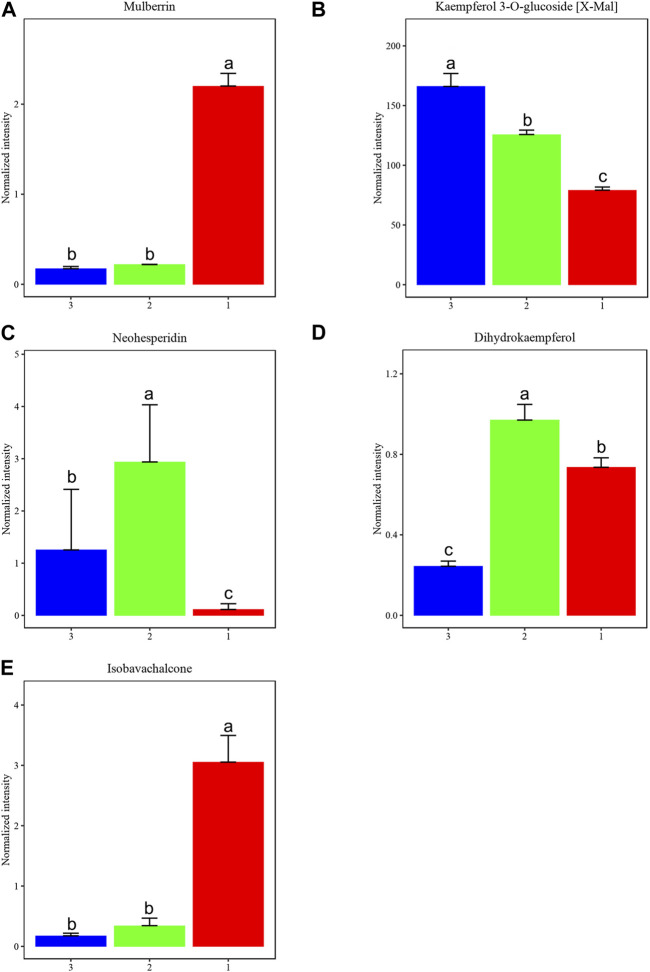
We identified key metabolites that differentiate among three groups of MLFs (CEP-red, RP-green, and NB-EP-blue) (n = 3). **(A)** Mulberrin, **(B)** Kaempferol 3-O-glucoside (X-Mal), **(C)** Neohesperidin, **(D)** Dihydrokaempferol, **(E)** Isobavachalcone. Columns with different superscript letters are significantly different (*p* < 0.05).

After filtering based on *p*-value ≤0.05 and VIP ≥1, along with one-way ANOVA with *p*-value ≤0.05 and VIP ≥1, we further analyzed key metabolites with biological significance among the three groups, providing research support for MLFs as exogenous antioxidant supplements. Figure 9 illustrates five significantly different compounds: mulberrin, kaempferol 3-O-glucoside [X-Mal], neohesperidin, dihydrokaempferol, and isobavachalcone among MLFs extracted using three methods.

Mulberrin and Isobavachalcone contents were significantly higher in CEP than in RP and NB-EP (Figure 9B). Neohesperidin and Dihydrokaempferol contents in RP were notably higher than in CEP and NB-EP (Figures 9C, D). Kaempferol 3-O-glucoside [X-Mal] contents in NB-EP exceeded those in CEP and RP (Figures 9A, E).

## 4 Discussion

High-intensity exercise in horses increases ROS levels, inducing oxidative stress and various pathologies (Mills et al., 1996). Implementing antioxidants is a common approach for ROS removal and alleviating oxidative stress (Kirschvink et al., 2008). Therefore, significant potential lies in exploring exogenous antioxidant supplements from botanical sources and studying their antioxidant mechanisms and collaborative impacts. Mulberry leaves, possessing medicinal and dietary attributes, offer wide-ranging applications. Within their constituents, MLFs exhibit diverse biological properties, including antibacterial, antioxidant, analgesic, anti-inflammatory, hypoglycemic, and lipid-lowering effects (Lee et al., 2014; Li et al., 2020; Duan et al., 2022; Zhang et al., 2022). Currently, utilizing active ingredients from traditional Chinese medicine in developing functional equine health products is a prominent trend.

H_2_O_2_-induced oxidative stress serves as the primary model for assessing the biological and antioxidant effects of natural products (Zhao et al., 2011; Park et al., 2022). H_2_O_2_ directly damages DNA, lipids, and other macromolecules, thereby propagating oxidative damage within cells and leading to cell death (Alía et al., 2005). Furthermore, H_2_O_2_ accumulation has been noted in equine sports and diseases, accompanied by cell apoptosis and mitochondrial dysfunction (Kirschvink et al., 2008; Goncalves et al., 2015; Ji et al., 2016). Our results demonstrated that ESMCs exposed to H_2_O_2_ showed elevated oxidative stress, decreased antioxidant capacity, and disrupted cellular energy metabolism, consistent with previous reports (Chodkowska et al., 2018a; b).

Skeletal muscle, due to its high metabolic activity, is prone to excessive free radical production and oxidative stress (Ji, 2007). Flavonoids, well-known antioxidants (Chagas et al., 2022), achieve their effects by neutralizing free radicals, triggering antioxidant enzymes, and thus alleviating oxidative stress and reducing metabolic products (Shen et al., 2022). Our study demonstrated that H_2_O_2_ treatment reduces GSH-Px and SOD activities, while increasing MDA levels. However, treatment with MLFs effectively reversed the decrease in GSH-Px and SOD activities and reduced MDA levels, enhancing the antioxidant status and improving cellular viability. This aligns with previous research, indicating that MLFs purified by AB-8 macroporous resin extraction increased the GSH-Px and SOD activities in INS-1 cells and reduced the cellular MDA content (Wang et al., 2019). Reduced MDA levels signified oxidative stress, which is crucial for ameliorating ESMC damage and enhancing cellular vitality. Interestingly, among the three MLFs types, the CEP exhibited the most significant efficacy in reducing MDA levels.

Previous studies have established a robust connection between flavonoids and the mitigation of oxidative stress through the Nrf2-ARE pathway in various cellular models (Kim et al., 2022). Specifically, flavonoid compounds in pre-treatments activate Nrf2 activity (Khan et al., 2020), which transcriptionally activate antioxidant and detoxifying enzymes, including SOD, glutathione peroxidase, and thioredoxin reductase (Yi et al., 2020; Kitaoka, 2021). We confirmed that all three MLFs pre-treatments effectively alleviated H_2_O_2_-induced oxidative stress in ESMCs by upregulating *Nrf2* gene expression, thereby activating downstream target genes *TrxR1*, *GSH-Px*, and *SOD*. Additionally, *Nelumbo nucifera* leaf flavonoid extract promotes *Nrf2* and *SOD-1* expression in liver cells under oxidative stress (Je and Lee, 2015). MLFs comprise diverse constituents, with each potentially regulating specific target genes within this pathway, resulting in varying gene expression levels in a dose-dependent way. These findings suggest that MLFs exert their antioxidant effects by inducing gene and antioxidant enzyme upregulation in ESMCs. Therefore, this complex process, involving multiple components, may explain their significant ability to enhance cell viability.

Mitochondria, serving as cellular powerhouses, generate over 90% of energy during equine locomotion (Dzięgielewska and Dunislawska, 2022). They are also primary sites for producing ROS. Excessive ROS accumulation can impair mitochondrial function, reducing efficiency and affecting muscular performance. Moreover, the survival of ESMCs in oxidative environments is an energy-demanding process that necessitates efficient metabolic adaptations to meet the bioenergetic demands, supporting cellular survival, proliferation, signaling, and environmental adaptation (Liu et al., 2019; Garrido-Pascual et al., 2020). Bioenergetics is central to environmental stress tolerance, demanding a delicate balance between energy input and expenditure for survival (Sokolova, 2013). Thus, ESMCs must adapt their metabolic processes for bioenergetic efficiency under adverse conditions. OCR and ECAR help quantify mitochondrial respiration and glycolytic function (Plitzko and Loesgen, 2018). We observed a markedly decreased mitochondrial respiratory capacity and glycolytic function in ESMCs exposed to H_2_O_2_. Conversely, pre-treatment with three MLF types enhanced mitochondrial respiratory capacity and glycolytic function. MLF pre-treatment enhances ESMCs’ adaptability to oxidative stress, potentially contributing to their high survival rate in such conditions. Mitochondrial biogenesis is an adaptive cellular stress response offering a promising therapeutic avenue for mitochondrial dysfunction and enhancing equine performance (Simmons et al., 2020). Various flavonoid compounds across categories have demonstrated the ability to induce mitochondrial biogenesis in diverse experimental models (Kicinska and Jarmuszkiewicz, 2020). By increasing the expression level of phosphorylated AMP-activated protein kinase (p-AMPK) and proliferator-activated receptor γ co-activator 1-α (PGC-α) proteins, MLFs promoted the translocation of glucose transporter 4 (GLUT4) to the cell membrane, thereby improving glucose uptake and utilization by L6 muscle cells and db/db mice. In addition, MLFs also enhanced the mitochondrial function of L6 muscle cells and db/db mice, which was reflected in the increased expression of nuclear respiratory factor 1 (NRF1), carnitine palmitoyl transferase 1 (CPT1) and cytochromic c oxidase subunit IV (COXIV) proteins, and the enhancement of mitochondrial membrane potential. As well as an increase in intracellular ATP levels and a decrease in ROS levels. (Meng et al., 2020). However, the mechanism behind the MLF-induced enhancement of cellular respiratory capacity of ESMC remains unclear and warrants further exploration.

Given the varying polarities of flavonoids, the extraction process selection is pivotal for maximizing the yield of active constituents (Garcia-Salas et al., 2010; Lin et al., 2022a). N-butyl alcohol, a highly polar organic solvent, demonstrates increased solubility for highly polar flavonoid compounds (Huang et al., 2022). Extracts with higher total flavonoid content exhibit superior biological activity and economic value (Peng et al., 2022). Our research findings indicate that utilizing butanol extraction in the purification of MLF products may be the optimal method for impurity removal and purity enhancement. Therefore, these findings align with previous research, indicating that the MLFs purified using D101 macroporous resin have higher purity than those obtained by the ultrasonic enzyme method (Zheng et al., 2022).

Flavonoids are classified into subclasses according to the heterocyclic C ring’s oxidation and unsaturation degree, as well as its connecting position to the aromatic B ring, yielding diverse derivatives (Guan and Liu, 2016; Wang et al., 2018; Shen et al., 2022). Previous research highlighted flavonols as the most copious flavonoids in food (Kumar and Pandey, 2013). MLFs contain numerous flavonol derivatives with potent antioxidant activity (Sugiyama et al., 2016), aligning with previous studies, as shown in Figure 1 (Garcia-Salas et al., 2010; Yang et al., 2014; Gryn-Rynko et al., 2016; Tam et al., 2021; Ma et al., 2022).

This study demonstrates MLFs’ ability to mitigate H_2_O_2_-induced oxidative stress in ESMCs. CEP, RP, and NB-EP demonstrated distinct pathways for alleviation and antioxidant properties. Therefore, we aimed to investigate the differential metabolites among these three MLF types. Flavonoid extraction depends significantly on solvent polarity, extraction methods, and duration, influencing the quantitative and qualitative composition of these compounds (Naeem et al., 2012). In this study, we employed ultrasound-assisted extraction, AB-8 macroporous resin purification, and n-butanol extraction techniques, enhancing the acquisition of target compounds with higher purification coefficients, including Mulberry, Kaempferol 3-O-glucoside (X-Mal), Neohesperidin, Isobavachalcone, and Dihydrokaempferol.

At the molecular level, MLFs, found in mulberry leaf extract, play a pivotal role in regulating cellular signal transduction pathways associated with antioxidants (Hu et al., 2022). The direct target of miR-337 is Nrf-2. Spinal cord injury can increase the level of miR-337 and cause oxidative stress, while mulberry flavonoids (Mulberrin) can reduce the expression level of miR-337, and improve the activity of antioxidant enzymes (including SOD and GSH) by regulating the upregulation of Nrf2. This reduces the high ROS levels caused by spinal cord injury (Xia et al., 2018). Moreover, Mulberrin treatment has been found to alleviate hepatic oxidative stress induced by intraperitoneal carbon tetrachloride injection in C57BL/6 N mice. Isobavachalcone demonstrates potent antioxidant activity by efficiently scavenging free radicals, including oxygen radicals (absorption capacity: 24.83 μM) and effectively eliminating hydroxyl radicals (Chen et al., 2002). Treating a Sephadex-induced rat lung injury model with Isobavachalcone ethyl ester reportedly upregulated the antioxidant enzymes GSH-Px and SOD, while reducing MDA (a lipid peroxidation product) (Gao et al., 2019). Kaempferol, the most common flavonol in plants, is typically present in various glycoside forms (Yonekura-Sakakibara et al., 2014; Hoang et al., 2015) and modulates cellular lipid and glucose metabolism (Hoang et al., 2015). Studies show that a diet high in flavonols, particularly kaempferol, is linked to higher GSH-Px and SOD levels in the body (Wang et al., 2015). Eohesperidin scavenges ROS (Hwang and Yen, 2008; Ortiz et al., 2022) and dose-dependently upregulates the expression of Nrf2 in SD rats (Wang and Cui, 2013). Dihydrokaempferol targets Keap1, which transcriptionally activates Nrf2, thereby ameliorating oxidative stress induced by caerulein and Lipopolysaccharide (LPS) (Liang et al., 2020). Most MLFs effectively mitigate H_2_O_2_-induced oxidative stress. Among them, Neohesperidin and Dihydrokaempferol in RP are the main players in regulating cellular respiration and exhibit higher antioxidant activity. Our study identified Mulberrin, Kaempferol 3-O-glucoside (X-Mal), Neohesperidin, Dihydrokaempferol, and Isobavachalcone as the principal bioactive flavonoids.

## 5 Conclusion

For this research, we evaluated the cytoprotective effects of three MLFs in activating the Nrf2/ARE pathway against H_2_O_2_-induced oxidative stress. Our findings reveal that MLFs pre-treatment effectively boosts SOD and GSH-Px expression in ESMCs via the Nrf2 pathway. Additionally, pre-treated cells show improved energy metabolism to counter oxidative stress. Among CEP, RP, and NB-EP, each exhibited distinct mitigation and antioxidant properties. CEP demonstrated the highest antioxidant activity, while RP and NB-EP exhibited low-dosage and high efficacy characteristics in alleviating oxidative stress in H_2_O_2_-induced ESMCs. Furthermore, we identified Mulberrin, Kaempferol 3-O-glucoside (X-Mal), Neohesperidin, Dihydrokaempferol, and Isobavachalcone as the primary bioactive flavonoids. In conclusion, MLFs represent potent exogenous antioxidant supplements for managing oxidative stress in exercising horses.

## Data Availability

The original contributions presented in the study are included in the article/supplementary material, further inquiries can be directed to the corresponding author.
